# Spinal cord *Schistosomiasis* in a 26-year-old male patient with progressive lower limb weakness and sensory loss: A case report

**DOI:** 10.1016/j.ijregi.2026.100867

**Published:** 2026-02-18

**Authors:** Ezekiel Baguma, Ivaan Pitua, Kenneth Nyombi

**Affiliations:** 1 School of Medicine, College of Health Sciences, Makerere University, Kampala, Uganda; 2 Department of Orthopedics, Mulago National Referral Hospital, Kampala, Uganda

**Keywords:** Spinal cord, *Schistosomiasis*, *Schistosoma*, Myelopathy, Praziquantel, Corticosteroids

## Abstract

Spinal cord *schistosomiasis* is a rare but potentially devastating manifestation of *Schistosoma* infection, resulting from an inflammatory granulomatous response to parasite eggs trapped in the spinal cord. It poses a significant diagnostic challenge, often mimicking intramedullary tumors. We report the case of a 26-year-old male patient residing near Lake Victoria who presented with acute, progressive paraplegia, sensory loss, and autonomic dysfunction. Magnetic resonance imaging revealed a focal enhancing intramedullary lesion at the T12-L1 level. Given the diagnostic uncertainty, a decompressive biopsy was performed, which confirmed the presence of *Schistosoma mansoni* ova within a granulomatous infiltrate. The patient was treated with a combination of praziquantel and systemic corticosteroids, resulting in significant neurologic recovery. This case highlights the critical importance of maintaining a high index of suspicion for *schistosomiasis* in patients presenting with myelopathy and relevant epidemiologic exposure. Prompt diagnosis, aided by histopathology or serology, and early initiation of dual medical therapy are essential to prevent permanent neurologic sequelae.

## Highlights

• Spinal cord schistosomiasis can closely mimic intramedullary spinal tumors.
• Epidemiologic exposure is key to early diagnosis of schistosomal myelopathy.
• Magnetic resonance imaging findings are suggestive but not diagnostic of spinal schistosomiasis.
• Combined praziquantel and corticosteroid therapy enables neurologic recovery.
• Histopathologic examination remains the gold standard for definitive diagnosis, particularly, when imaging findings mimic intramedullary tumors or other inflammatory myelopathies.

## Introduction

Spinal cord *schistosomiasis* (SCS) is a rare yet potentially debilitating manifestation of *Schistosoma* infection, predominantly affecting individuals in endemic regions [1,2]. Human *schistosomiasis* remains a significant global health challenge, with endemic transmission documented in 78 countries. Although the disease is widespread in tropical and subtropical regions, sub-Saharan Africa bears the highest burden, accounting for approximately 84.25% of all global infections [3]. Within these endemic regions, disease prevalence exhibits significant localized variation, primarily dictated by proximity to freshwater bodies, such as lakes, rivers, and irrigation dams, which serve as habitats for the intermediate snail host [4].

Systemically, *schistosomiasis* typically manifests in three stages: an initial cercarial dermatitis (“swimmer’s itch”) at the site of skin penetration, followed by acute *schistosomiasis* (Katayama fever), a systemic hypersensitivity reaction characterized by fever, malaise, and hepatosplenomegaly [5]. Chronic infection often progresses to gastrointestinal, hepatosplenic, or urogenital disease, depending on the species [[6], [7], [8]].

In contrast, SCS represents a distinct neurologic entity because pathophysiology involves the migration of *Schistosoma* eggs to the spinal cord. Once the eggs are deposited in the neural tissue, they do not simply remain passive; rather, they trigger a granulomatous inflammatory response [9]. This host immune response is critical to the disease process. The formation of granulomas around the eggs leads to space-occupying lesions and associated inflammation that can cause irreversible damage if left untreated [6,9]. The inflammation results in compression and destruction of the delicate spinal cord tissue, leading to the clinical signs of myelopathy. This process highlights the destructive nature of the immune reaction to the parasite eggs within the confined space of the spinal canal [6,10].

Despite its rarity, SCS should be considered in patients with progressive myelopathy who have a history of exposure to endemic areas [2,11]. The clinical presentation of SCS frequently mimics other acute or subacute myelopathies, necessitating a broad differential diagnosis. Key conditions that present with similar neurologic deficits include idiopathic transverse myelitis, spinal tuberculosis (Pott’s disease), and intramedullary neoplasms such as ependymomas and low-grade gliomas [10,12]. Distinguishing SCS from these mimics relies on specific biomarkers; although SCS is often associated with peripheral blood eosinophilia and positive anti-*Schistosoma* serum immunoglobulin (Ig)G/IgM antibodies, these inflammatory markers are typically absent in neoplastic processes [13]. Furthermore, unlike the focal granulomatous enhancement seen in SCS, biomarkers for transverse myelitis may include specific cerebrospinal fluid oligoclonal bands or aquaporin-4 antibodies, depending on the underlying etiology [14]. Therefore, early recognition and intervention are crucial to prevent permanent neurological disability. The window for effective treatment is often limited by the progression of the inflammatory response. We report a case of a 26-year-old male patient with spinal *schistosomiasis* who presented with progressive lower limb weakness, sensory loss, and bladder dysfunction.

## Case presentation

A 26-year-old, previously healthy male presented with a 2-week history of progressive lower limb weakness and numbness. Symptoms began with severe, localized lower back pain associated with difficulty walking and rapidly progressed over 1 week to complete inability to stand or ambulate. This was accompanied by bilateral lower limb sensory loss and loss of bladder and bowel control. He denied antecedent trauma, fever, recent infection, or other systemic symptoms.

The patient reported a history of frequent contact with the waters of Lake Victoria at the Ggaba landing site in Kampala, where he resided. Although local data for Ggaba is limited, the Lake Victoria shoreline is well-documented as a hyper-endemic zone for *Schistosoma mansoni,* reportedly having one of the highest burdens of the parasite worldwide [15]. He had no significant past medical history and no previous neurologic symptoms. The rapid progression of neurologic deficits raised concern for an acute compressive or inflammatory spinal cord pathology.

On examination, the patient was clinically stable with normal vital signs (blood pressure 126/82 mmHg, pulse rate 78 bpm, respiratory rate 18 cycles/min, and temperature 36.7°C). There was no pallor, jaundice, lymphadenopathy, or other signs of systemic illness. General and systemic examinations were unremarkable.

Neurologic examination revealed flaccid paraplegia, with motor power graded 0/5 in both lower limbs. Sensory testing demonstrated reduced pinprick and light touch sensation bilaterally, extending from below the knees to the mid-thigh level. Deep tendon reflexes, including patellar and Achilles reflexes, were markedly reduced bilaterally. Examination of cranial nerves, upper limb motor and sensory function, and coordination was normal, localizing the lesion to the thoracolumbar spinal cord.

## Investigations

Laboratory investigations revealed a normal complete blood count, except for marked eosinophilia (white blood cells 7650/µl [4000-11,000], eosinophils 16% [1-4%]), hemoglobin (15.6 g/dl [[11], [12], [13], [14], [15], [16], [17]]), platelet count (315,000/µl [150,000-400,000]). Comprehensive metabolic paneling showed normal serum electrolytes, including sodium (138 mmol/l [135-145 mmol/l]), potassium (4.1 mmol/l [3.5-5.1 mmol/l]), and chloride (102 mmol/l [98-107 mmol/l]). Liver function tests were also within normal limits, with an alanine aminotransferase of 32 U/l (<41 U/l), aspartate aminotransferase of 28 U/l (<40 U/l), and total bilirubin of 0.8 mg/dl (0.1-1.2 mg/dl), suggesting no overt hepatosplenic involvement at the time of presentation. Serological testing was positive for IgG and IgM antibodies against *Schistosoma mansoni*. Plain radiographs of the thoracolumbar spine were unremarkable.

**Figure 1:**
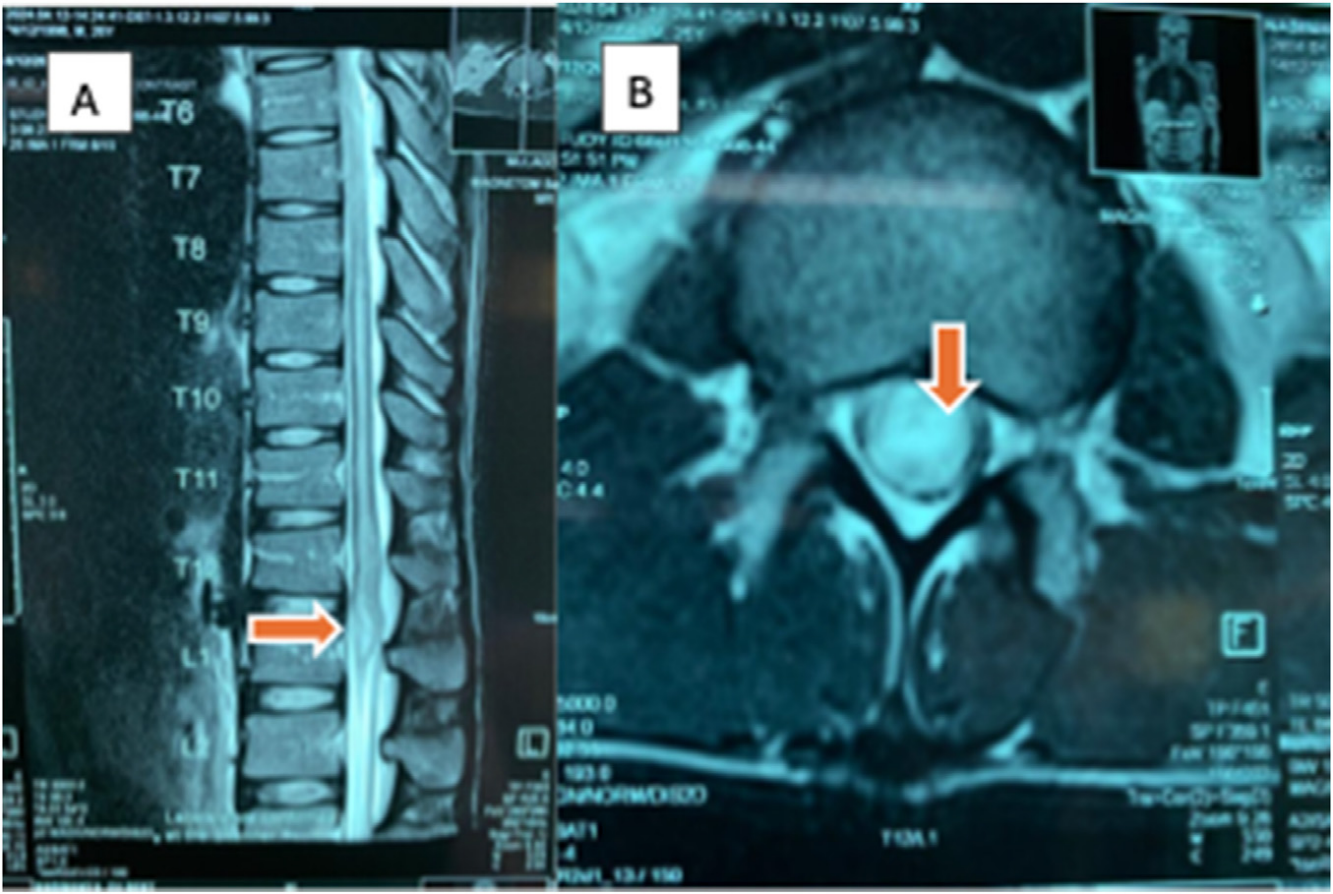
T2-weighted magnetic resource imaging (a: sagittal and B: axial views) showing a focal intramedullary lesion (arrows) at the T12-L1 level.

Magnetic resonance imaging (MRI) of the thoracolumbar spine with gadolinium contrast demonstrated a focal intramedullary lesion at the T12-L1 level, characterized by hyperintensity on T2-weighted images with contrast enhancement (Figure 1). These findings, together with eosinophilia, positive serology, and epidemiologic exposure, strongly suggested SCS.

Given the progressive neurologic deterioration, a decompressive biopsy at the L1 level was performed. Histopathologic examination revealed collapsed *Schistosoma* ova surrounded by granulomatous inflammation and dense eosinophilic infiltrates, confirming SCS.

## Management and follow-up

The patient was commenced on praziquantel therapy (1200 mg, single dose) for antiparasitic treatment, alongside systemic corticosteroids (intravenous dexamethasone 8 mg given 8-hourly for 3 days) to reduce spinal cord inflammation and prevent further neurological injury.

Neurologic improvement was observed within 1 week of treatment initiation, with return of motor activity graded 1/5 in the hip flexors and extensors and partial sensory recovery. The patient was discharged on a supervised physiotherapy program.

At 1-month follow-up in the spine outpatient clinic, follow-up laboratory investigations at showed a normalization of the absolute eosinophil count to 240/µl. No other further workup was done; the patient had significant clinical and functional recovery. Motor strength had improved to 3/5 in the hip flexors and extensors. He was able to sit independently in a wheelchair and reported marked improvement in lower limb sensation, indicating a favorable early neurologic recovery.

At 6-month follow-up, motor strength further improved to the power of 4/5 in the lower limbs and the patient was able to ambulate using axillary crutches and fully control his bowel and bladder.

## Discussion

This case illustrates a classic presentation of SCS in a young male from an endemic region, emphasizing the critical importance of epidemiologic history in diagnosing acute myelopathy. Although *Schistosoma* infection is widespread in sub-Saharan Africa, spinal cord involvement remains a rare but severe complication, occurring when parasite eggs are embolized via the valveless Batson’s venous plexus into the spinal vasculature [8,16]. Once deposited in the spinal cord, the eggs incite a granulomatous inflammatory response, leading to space-occupying lesions, edema, and subsequent compression of neural tissue, which manifests clinically as myeloradiculopathy [10].

Diagnosing SCS is often challenging because its clinical and radiological features frequently mimic intramedullary tumors, such as ependymomas or gliomas, as well as other inflammatory conditions such as transverse myelitis [2]. In the presented case, the rapid progression of paraplegia and the presence of a focal enhancing lesion on MRI initially raised concerns for a malignancy. Recent literature highlights that although MRI findings (typically T2 hyperintensity with nodular or arborized enhancement) are suggestive, they are not pathognomonic [1]. Consequently, definitive diagnosis often requires histopathologic confirmation, particularly, when clinical features are ambiguous or systemic signs of *schistosomiasis* are absent [17]. The histopathologic identification of collapsed S. mansoni ova, characterized by their distinct lateral spines and surrounded by intense eosinophilic granulomatous inflammation, serves as the definitive diagnostic feature in this case. This finding confirms that the patient’s neurologic deterioration was not a result of direct parasitic invasion of neural tissue but rather a manifestation of an exuberant type IV hypersensitivity response to the highly antigenic eggs trapped within the spinal vasculature. This inflammatory cascade leads to focal mass effect, localized edema, and eventual axonal destruction, explaining why the MRI findings initially mimicked the infiltrative growth pattern of a primary intramedullary malignancy [18]. The presence of these granulomas provides the pathological basis for the patient’s rapid clinical response to corticosteroids, which served to dampen this immune-mediated injury, alongside the parasiticidal action of praziquantel.

The management of SCS relies on a combination of antiparasitic therapy and corticosteroids. Praziquantel remains the gold standard for eliminating adult worms and halting egg production, whereas systemic corticosteroids are essential to suppress the hypersensitivity reaction and reduce granuloma-associated edema [1,2,10,16,17]. The decision to perform a decompressive biopsy in this patient facilitated a definitive diagnosis and likely contributed to resolving the mass effect. This patient’s favorable outcome, characterized by the return of motor function and sphincter control, reinforces the reversibility of neurologic deficits when treatment is not delayed.

## Implications to clinical care

SCS highlights the critical need to include *schistosomiasis* in the differential diagnosis of patients presenting with progressive weakness, sensory deficits, and sphincter dysfunction, particularly, when there is a history of residence in or travel to endemic regions. The disease closely mimics other spinal cord pathologies, and failure to consider a parasitic etiology may result in diagnostic delay and permanent neurologic disability. A thorough epidemiologic history should, therefore, be a routine component of neurologic assessment in appropriate contexts. Early recognition and timely treatment with praziquantel and corticosteroids can significantly improve outcomes and prevent irreversible neurologic damage.

## Conclusion

The presentation of this 26-year-old male patient highlights the diagnostic complexity of SCS, a condition that frequently masquerades as a spinal neoplasm or transverse myelitis. The pathophysiology is driven by the host’s immune response to eggs deposited via the valveless vertebral venous plexus, necessitating a treatment strategy that addresses the parasite and the inflammation. The patient’s favorable outcome after decompressive biopsy and dual therapy with praziquantel and corticosteroids reinforces that neurologic deficits are reversible if treated within the acute window. Clinicians practicing in or treating patients from endemic regions must include SCS in the differential diagnosis of acute myelopathy to avoid delays in management that could lead to irreversible disability.

## Declaration of competing interest

The authors have no competing interests to declare.
